# Automated analysis of phylogenetic clusters

**DOI:** 10.1186/1471-2105-14-317

**Published:** 2013-11-06

**Authors:** Manon Ragonnet-Cronin, Emma Hodcroft, Stéphane Hué, Esther Fearnhill, Valerie Delpech, Andrew J Leigh Brown, Samantha Lycett

**Affiliations:** 1 University of Edinburgh, Edinburgh, UK; 2 University College London, London, UK; 3 MRC Clinical Trials Unit, London, UK; 4 Public Health England, London, UK

**Keywords:** Phylogenetics, Cluster, Sequence analysis, Virus, HIV, Epidemiology

## Abstract

**Background:**

As sequence data sets used for the investigation of pathogen transmission patterns increase in size, automated tools and standardized methods for cluster analysis have become necessary. We have developed an automated Cluster Picker which identifies monophyletic clades meeting user-input criteria for bootstrap support and maximum genetic distance within large phylogenetic trees. A second tool, the Cluster Matcher, automates the process of linking genetic data to epidemiological or clinical data, and matches clusters between runs of the Cluster Picker.

**Results:**

We explore the effect of different bootstrap and genetic distance thresholds on clusters identified in a data set of publicly available HIV sequences, and compare these results to those of a previously published tool for cluster identification. To demonstrate their utility, we then use the Cluster Picker and Cluster Matcher together to investigate how clusters in the data set changed over time. We find that clusters containing sequences from more than one UK location at the first time point (multiple origin) were significantly more likely to grow than those representing only a single location.

**Conclusions:**

The Cluster Picker and Cluster Matcher can rapidly process phylogenetic trees containing tens of thousands of sequences. Together these tools will facilitate comparisons of pathogen transmission dynamics between studies and countries.

## Background

In order to control the spread of disease and optimize public health interventions, it is crucial to understand how transmission from one individual to the next occurs. Identifying at risk individuals and behaviors through contact tracing has been a successful strategy in controlling many infectious diseases. Recently, the rise of sequencing and other technologies have meant that disease transmission can be studied at the molecular level. One example of molecular epidemiology is the reconstruction of transmission trees based on the genetic relatedness of pathogens, which reflect the relationships between infected individuals [1].

With their fast evolving genomes [2], RNA viruses are particularly well suited to phylogenetic analyses, and studies have been carried out extensively on HIV [1,3,4], as well as on hepatitis C [5], Ebola [6], severe acute respiratory syndrome (SARS) [7] and dengue [8]. Despite their lower genetic diversity, phylogenetic analyses are increasingly being used to investigate the transmission of DNA viruses such as herpes and even of bacteria [9,10].

Due to the uncertainty in infection time, evolutionary rate and potential contacts, it is generally not possible to reconstruct the exact transmission network from a phylogenetic tree alone. However patients sharing similar viruses are potentially epidemiologically linked, so local outbreaks within the larger epidemic can be identified by finding transmission clusters. Clusters in epidemiology are broadly described as an unusual aggregation of infection, perceived to be greater than that expected by chance. In networks, clusters are quantitatively defined as a group of nodes having a local clustering coefficient significantly greater than that of a random graph with the same number of vertices and the same mean shortest path [11]. In a phylogenetic tree, clusters contain sequences from different patients which share a recent common ancestor. These clusters are manifest as groupings in the phylogenetic tree in which we have high confidence and which are likely to reflect recent or ongoing transmission. However, defining and detecting meaningful transmission clusters from a population sample in a phylogenetic tree is not straightforward, and various strategies have been proposed and used in the literature.

Clusters are often defined based on high support (bootstrap or posterior probability) and/or low within cluster genetic distance, but the thresholds for both vary. For HIV, bootstraps ranging from 70% and up to 99% have been used [5,12-16], in combination with within-cluster genetic distances from 1% to 4.5% substitutions per site [3,13-15,17,18]. The method for calculating within cluster genetic distance also varies: the mean of the pairwise genetic distances of clustered sequences has been employed [16], as well as their median [19]. Another alternative is “single linkage”, where a sequence is included in a cluster if its distance to just one other sequence in the cluster is below the threshold [20,21]. If time resolved trees are used (which require knowledge or inference of a molecular clock), clusters can be defined based on time to most recent common ancestor [22]. These most resemble clusters generated using maximum genetic distance in a non-time resolved distance-based tree.

In the case of HIV, analyses of phylogenetic clusters have been used to identify correlates of transmission including risk group [18], stage of infection [23,24], cluster size [25], the presence or absence of co-infections, including other sexually transmitted infections [13] as well as drug treatment and compliance. A recent study used a phylogenetic approach to determine the relative contribution of each of these variables to the risk of onward transmission [26], finding that antiretroviral treatment decreased HIV transmission risk.

With sequence data sets used for the reconstruction of phylogenies now containing tens of thousands of sequences, identifying clusters manually is infeasible. Using in-house pipe lines for detecting clusters is possible, but in order to compare results between studies, freely available software tools would be advantageous. Based on the support and genetic distance criteria commonly used, we have developed the Cluster Picker (CP) which identifies clusters in phylogenetic trees. Furthermore, we introduce the Cluster Matcher (CM), the first tool to describe identified clusters epidemiologically as well match clusters between phylogenetic trees. To demonstrate their utility, we use both these tools to examine subtype B cluster dynamics in the UK and we compare CP performance to that of other available software.

## Implementation

The Cluster Picker and Cluster Matcher have been developed in Java 1.6 and are platform-independent. Both programs can be downloaded freely from http://hiv.bio.ed.ac.uk/software.html as functioning jar files with accompanying tutorials, manuals and test files. Source code is available on Google code (http://code.google.com/p/cluster-picker-and-cluster-matcher/) under GNU GPLv3.

### The Cluster Picker

#### Objective

The CP is a JAVA based program that identifies clusters of sequences in a phylogenetic tree based on support for the node (bootstrap or posterior probability) and the maximum pairwise genetic distance within the cluster.

#### Input

The CP takes as input a set of aligned sequences in fasta format and a newick tree built from those same sequences, with support values on the nodes. The user inputs the desired node support threshold and maximum genetic distance for clusters, as well as an initial support threshold for splitting the tree prior to analysis.

#### Algorithm

The CP utilizes a depth-first algorithm to explore the tree: starting at the root and working its way along each branch before backtracking when a leaf is reached. In order to minimize the number of pairwise distances computed (thus reducing running time), the tree is initially split. The user inputs an initial node support threshold, and starting from the root, the tree is divided into subtrees supported at this threshold. Further analyses will take place only within these subtrees; therefore, the initial support threshold must necessarily be smaller than or equal to the cluster support threshold. Starting from the root of the subtree, the CP proceeds to the first node exceeding the bootstrap support threshold. All sequences within the group are identified and their pairwise genetic distances are calculated. If the largest of these is smaller than or equal to the user-input maximum genetic distance threshold, the group of sequences is identified as a cluster. If the maximum pairwise distance is larger than the threshold, the cluster is rejected and the algorithm proceeds to the next supported node and repeats the same analysis. When a leaf is reached, the CP backtracks to the last node whose children have not been fully analyzed. When the algorithm has analyzed the entire tree, a list of clusters matching the user-input criteria is generated. Note that because the algorithm proceeds from the root towards the tips, nested clusters are not identified and do not appear in the final list.

#### Output

The CP outputs a log file listing for each cluster: cluster number, cluster size, maximum genetic distance within the cluster, support value and tip names. Also output are a fasta file in which sequence names are preceded by their cluster number and two trees, one in newick format and one in FigTree format (http://tree.bio.ed.ac.uk/software/figtree/). In both trees sequence names are preceded by cluster name, and in the FigTree file, sequence names are colored by cluster.

### The Cluster Matcher

#### Objective

The CM is a JAVA based program which links clusters output by different CP runs based on the names of sequences within them. This can be done for CP runs on the same dataset, for example to examine changes following a change in method, or after the addition of new sequences to the dataset. The CM can also be used to identify clusters that meet certain criteria in double or single data set mode, outputting FigTree format files for each cluster. On top of this, the CM outputs a description of each cluster, for example summarizing epidemiological data associated with clustered sequences.

#### Input

The CM takes as input the newick files output by the CP and, as an option, corresponding annotation files. Inputting an annotation file allows the user to select clusters based on those annotations. For example, if the annotation file contains risk group data, the CM could output only clusters containing at least 50% of sequences from men who have sex with men (MSM). The user can also choose to output clusters based on whether they contain a specified minimum number of sequences.

#### Algorithm

Traversing from root-to-tip, the CM first identifies all clusters present in each dataset, linking every sequence in a cluster to any epidemiological information provided. The CM then examines clusters present in the first data set to determine if the sequences are clustered in the second data set. In this manner, each cluster from the first data set is linked to clusters in the second data set that contain matching sequences, and vice versa. For each cluster, information is retrieved including its size, number of matching sequences, and the distribution of epidemiological traits attached to its sequences. This allows the clusters to be easily filtered when the user specifies cluster selection criteria, and is used to generate summary information for each cluster.

#### Output

The CM outputs a FigTree file for each matching pair of clusters (or each cluster if used in single data set mode) that is consistent with user specifications, as well as a log file detailing settings and summarizing results. The FigTree file contains four trees showing the matched clusters in both trees, and a zoom into each of those clusters, allowing for the visualization of single clusters within large phylogenies.

## Results

### Data

Publicly available HIV *pol* sequences from the UK HIV Drug Resistance Database (HIVRDB; http://www.hivrdb.org) were used to evaluate the Cluster Picker and Cluster Matcher (Genbank IDs: EU236439–EU236538 [3], GQ462027-GQ462532 [18], JN100661–JN101948 [22]). Sequences were subtyped in Rega (http://dbpartners.stanford.edu/RegaSubtyping/), and cover the entire protease gene and up to 900 bases of reverse transcriptase. Sequences were stripped of 45 sites associated with drug resistance based on the 2011 updated drug resistance list [27]. In parallel, all unique subtype B *pol* sequences (HXB2 coordinates 2253 to 3549) with no insertions or deletions were downloaded from the Los Alamos National Laboratory HIV Sequence Database (http://www.hiv.lanl.gov) in order to perform speed comparison between the Cluster Picker and PhyloPart. Viral datasets for hepatitis C virus and influenza (avian, pandemic and seasonal) are analyzed in Additional file 1.

### Effect of cluster thresholds on cluster distribution

Using the CP, we evaluated the effect of different cluster thresholds for genetic distance and cluster support on cluster identification among the UK subtype B sequences downloaded. One hundred replicate alignments were generated and a maximum likelihood tree with bootstraps was reconstructed in FastTree v2. 1. 4 [28] with a subtype C reference sequence (GenBank accession number: AY772699). The Cluster Picker runs on newick format trees generated in any program, as shown in Additional file 1.

**Figure 1:**
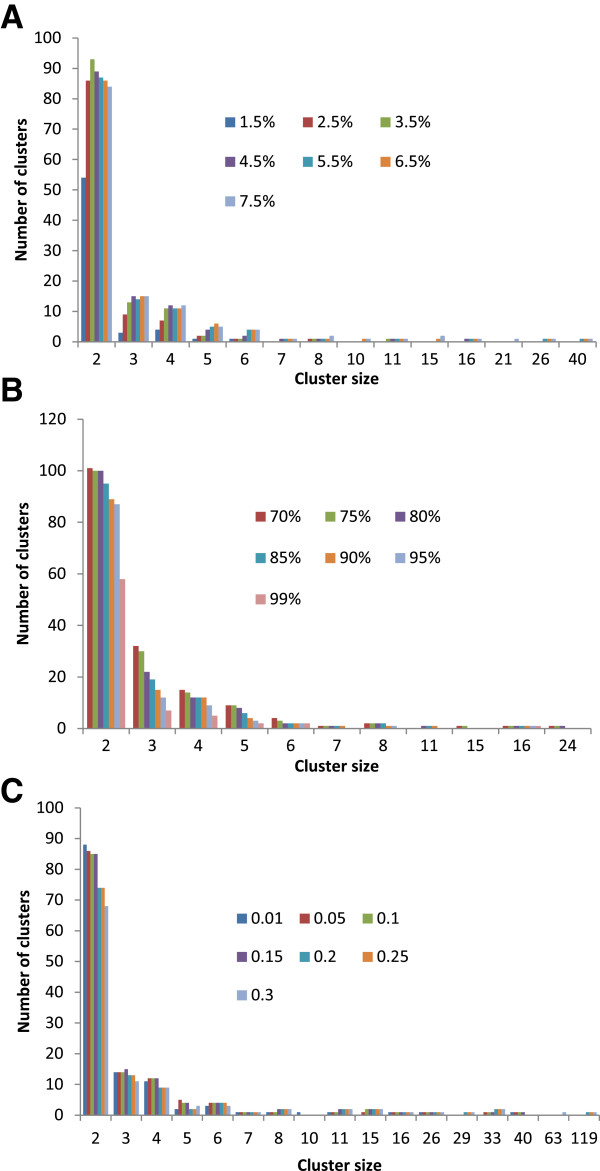
**Cluster distributions.** 1381 subtype B UK sequences from NCBI were processed **(A)** through the Cluster Picker, with bootstrap support threshold fixed at 90% and maximum genetic distance threshold varied between 1.5% and 7.5%, **(B)** through the Cluster Picker with maximum genetic distance threshold fixed at 4.5% and bootstrap support threshold varied between 70% and 99%, and **(C)** through PhyloPart, with the t-percentile threshold varied between 1% and 30%.

**Figure 2:**
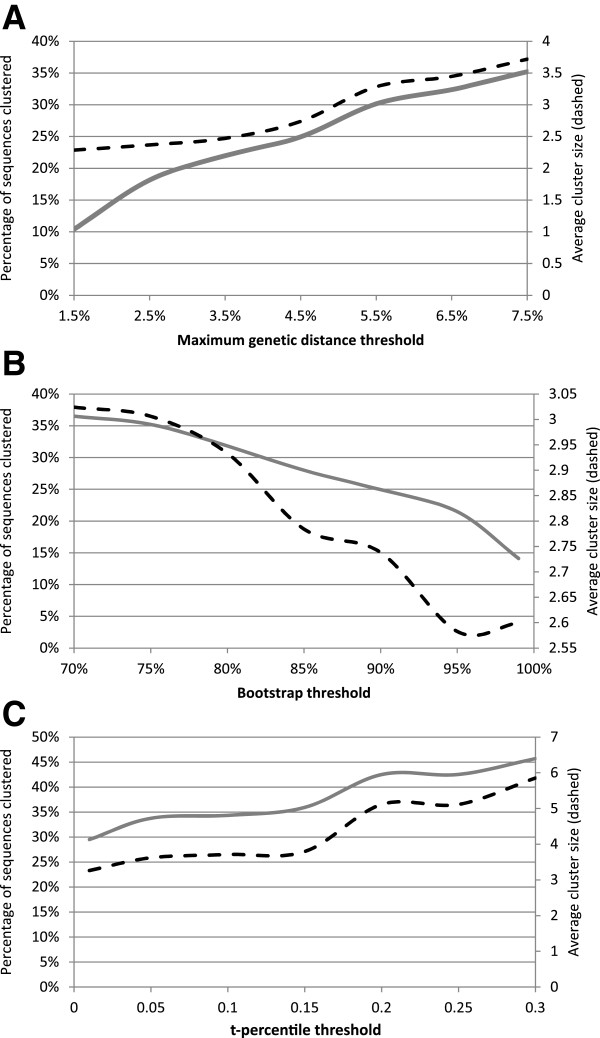
**Clustering patterns.** 1381 subtype B UK sequences from NCBI were processed **(A)** through the Cluster Picker, with bootstrap support threshold fixed at 90% and maximum genetic distance threshold varied between 1.5% and 7.5%, **(B)** through the Cluster Picker with maximum genetic distance threshold fixed at 4.5% and bootstrap support threshold varied between 70% and 99%, and **(C)** through PhyloPart, with the t-percentile threshold varied between 1% and 30%. Distribution for varying bootstrap thresholds. For each threshold, we plotted the percentage of total sequences in clusters (grey line) and average cluster size (dashed line).

Of 1831 downloaded sequences, 1381 unique subtype B sequences were used to examine the effect of cluster definition on cluster distribution using the CP. Although the phylogenetic tree contained a reference subtype C sequence, this outgroup was removed prior to analysis with the CP using the APE package v.3.0-8 in R [29,30]. Initially, we fixed the bootstrap threshold in the CP at 90% and varied within-cluster maximum genetic distance between 1.5% and 7.5%. Between 4.5% and 7.5%, we found that for the most part, the same clusters were identified (Figure 1A). Within this range, the number of clusters stabilized around 128 (ranging from 126 to 131), with 2/3 containing only two sequences. At a genetic distance of 1.5%, only 63 clusters were identified. The proportion of sequences in clusters and average cluster size both increased as the genetic distance threshold was increased (Figure 2A). At a maximum genetic distance of 4.5%, 25% of sequences clustered, identical to the proportion found after a time-resolved analysis of the same sequences [3]. Beyond 4.5%, the ratio of these two measures became constant, indicating that as the genetic distance cut-off was relaxed sequences were being added equally to all clusters. The effect of varying the cluster bootstrap threshold was different; fixing the genetic distance at 4.5%, the proportion of sequences in clusters decreased gradually as bootstrap thresholds were increased from 70% to 99% (Figures 1B and 2B).

### Automated analysis of cluster dynamics

**Figure 3:**
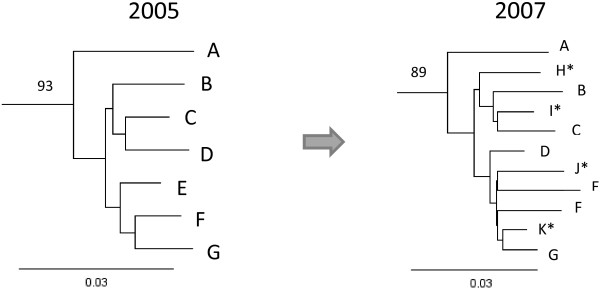
**Dynamics of a single cluster 2005-2007.** In this example, the cluster identified in 2007 no longer matches the initial cluster definition as bootstrap support has dropped from 93% to 89%. Sequences **A** to **G** are those already in the cluster in 2005, starred sequences **(H to K)** have been added to the cluster in the intervening years.

Using both the CP and the CM we reconstructed cluster dynamics over time, analyzing 409 non-B UK sequences as well as the 1381 subtype B sequences. These included 63 A subtypes, 219 C and 127 other non-B. All 1790 anonymised sequences had linked sampling date and location information in the HIVRDB. A phylogenetic tree was initially constructed from 1212 sequences of all subtypes collected up to 2005. A total of 148 clusters, containing 431 sequences (35.6%), were supported by a bootstrap ≥90% and had a maximum genetic distance ≤4.5%. One hundred and eight of these clusters were pairs, while the largest contained seventeen sequences. A second tree was built from the entire dataset of 1790 sequences and clusters matched between the early and late trees so that cluster changes could be described. In support of our initial cluster definition, the genetic distance of the new clusters increased above 4.5% only in two clusters despite the addition of 578 sequences, while bootstrap dropped below 0.90 only for six clusters (Figure 3). Finally, each clustered sequence was linked to sample location information in the HIVRDB and the CM was used to sort clusters in 2005 based on whether they contained sequences from a single sample location (“single” origin) or more (“multiple” origin). The UK HIV Drug Resistance Database categorizes geographical origin into 17 areas, all of which were represented in this dataset. A large proportion of sequences originate from the London area (one center). Patterns of change of single origin versus multiple origin clusters were compared (R script available in Additional file 2) [29]. Of 148 clusters, 63 were thus classified as multiple origin and 85 as single origin (Additional file 3). For each cluster, cluster growth was then calculated as the number of new sequences per initial sequence [25]. Mean cluster growth differed significantly between single and multiple origin clusters (0.155 vs. 0. 302, respectively, Kruskal-Wallis test: p = 0.0016; Additional file 2).

### Comparison with PhyloPart

**Figure 4:**
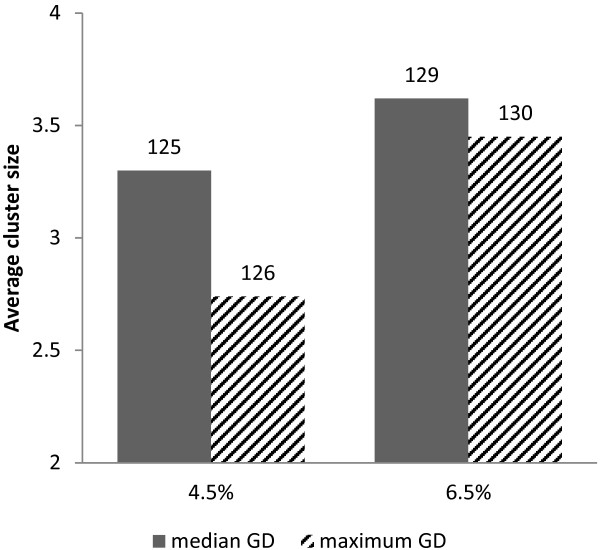
**Average cluster size according to clustering method.** At thresholds of 4.5% and 6.5%, PhyloPart (in grey, median GD) and the Cluster Picker (dashed, maximum GD) identified nearly exactly the same number of clusters (numbers above the columns) but PhyloPart clusters were on average larger. GD genetic distance.

We wished to compare the performance of the CP to PhyloPart, a recently released software tool for the identification of clusters [19]. PhyloPart generates the pairwise distance distribution for a tree and identifies a group of sequences as a cluster if the median of their genetic distances is below a user-input t-percentile threshold of the whole-tree distance distribution. The rooted subtype B tree containing 1381 sequences was analyzed in PhyloPart, varying the t-percentile threshold for cluster identification from 1% to 30%. Upon examination of the output, it appeared that this range reflected median genetic distances within clusters from 4.5% to 9% in the data. Once again, cluster distribution was not very much affected by the cut-off (Figure 1C), but the proportion of sequences in clusters and average cluster size increased as cluster definition was relaxed (Figure 2C). As a t-percentile threshold of 0.01 and 0.05 corresponded to genetic distance cut-offs of 4.5%, and 6.5%, respectively, the CP and PhyloPart output were compared in more depth at each of these two matched thresholds. Each time, the number of clusters and the cluster distributions were near identical (KS test, p = 0.9998 and p = 1 for 4.5% and 6.5% respectively). However, as expected, individual cluster sizes were significantly reduced when maximum within cluster genetic distance was used instead of median (Figure 4; one-sample sign test, p = 6.1*10^-5^ and p = 0.03 for genetic distances of 4.5% and 6.5%, respectively).

In order to make the comparison we ran both the CP and PhyloPart on 18 data sets sized 1000 to 18,000. A maximum likelihood tree with bootstraps was initially built from 18,000 sequences downloaded from Los Alamos. Then, sets of 1000 tips were dropped sequentially from the tree to generate trees with variable number of tips (Additional file 2). As PhyloPart does not print time to completion, it was launched from within a python script with an additional function to calculate running time (Additional file 4). Both programs were able to process trees with up to 17,000 sequences in less than an hour on a desktop (Table 1, Additional File 5), although PhyloPart did not terminate on the largest dataset (n = 18,000 sequences). The CP completed on average three times faster than PhyloPart.

**Table 1:** Time to completion (in seconds) of the Cluster Picker and PhyloPart for data sets of increasing sizes

**Number of sequences**	**Cluster Picker (s)**	**PhyloPart (s)**
1000	13.098	8.913
2000	36.137	44.151
3000	68.772	112.729
4000	115.618	672.085
5000	173.584	1447.047
6000	244.290	1713.749
7000	328.651	2190.336
8000	419.369	1081.785
9000	526.070	1043.838
10000	658.607	2321.955
11000	769.469	2343.197
12000	911.086	3061.134
13000	1059.509	2851.417
14000	1228.151	2078.609
15000	1383.366	2625.491
16000	1581.351	2797.329
17000	1775.639	3047.713
18000	1990.372	NA

Notes: Both programs were run on a Windows desktop computer with an Intel Core i5-2400 3.10 GHz with 4 processors, reserving 1.5 G of heap space. PhyloPart did not complete on the desktop computer with n = 18,000 sequences as heap space could not be increased. Settings were left as default in the Cluster Picker and set at t = 0.05 in PhyloPart. For 10,000 sequences, program specific RAM usage was 265,000 K for PhyloPart and 100,000 K for the CP. Computational complexity approximately O(n^2^) for this data set (see Additional file 5).

## Discussion

The tools that we present here can be used to investigate the dynamics of pathogen transmission. The CP is able to rapidly identify clusters in an automated way in large datasets, based on criteria demonstrated previously to accurately delineate epidemiologically relevant clusters [16]. Because in many cases cluster studies seek to combine genetic with epidemiological or clinical data (such as risk group or stage of infection), we have also made available the CM, which links clusters between runs and to epidemiological data. In contrast to some other methods available for the analysis of trait-annotated phylogenies [31,32], the CM does not require any assumptions to be made about the heritability of the traits examined, as it does not look for associations between the distribution of traits and the phylogeny, only summarizes their distribution. As an example, we used the tools together to investigate the dynamics of single vs. multiple origin HIV clusters in the UK, as well as conduct preliminary analyses of HCV and influenza clustering.

There was remarkable consistency in the clusters identified at maximum genetic distances between 4.5% and 7.5%, as has been previously observed [3]. We conclude that these clusters represent well-delineated epidemiological units in the UK HIV epidemic. In contrast, when the maximum genetic distance threshold was decreased to 1.5%, only half of the clusters were identified. These clusters defined by such a short distance will reflect recent transmissions and frequent samplings [17]. In contrast, the UK HIVRDB contains mostly sequences from chronically infected patients, many of whom were first sequenced long after infection, and so in order to identify relevant clusters, a threshold of 4.5%, as we have used before [3,18], is more appropriate. The effect of the bootstrap threshold was less evident, and so we conclude that genetic distance is the key parameter for epidemiologically relevant clustering. We stress however that the present analysis alone is not sufficient to yield a reusable definition of cluster threshold parameters, as the data set of publicly available sequences was too small for extensive testing. To resolve this issue, we are currently conducting in depth analyses on the UK HIVRDB as a whole (<50,000 sequences). Using the CP many thresholds can be examined very rapidly.

In order to highlight the CP’s suitability to other viruses and epidemic patterns, we conducted analyses of HCV and three datasets of influenza sequences (see Additional file 1). The CP was able to pick out meaningful pandemic flu clades consistent with earlier work [33], and the analysis of ladder-like seasonal influenza demonstrated the CP can accommodate different tree shapes, with sequences from the same year clustering together.

The CP uses maximum within cluster genetic distance, while PhyloPart, another recently released sequence clustering tool, uses the median. In previous studies, we have identified clusters in trees based on mean within cluster distance [18,22]. However, we decided to use maximum genetic distance in our tool for three reasons. First, maximum genetic distance (as well as median genetic distance) is less affected by the number of sequences within a cluster (which can be the result of more or less intensive population sampling and contact tracing). When the mean is used, the distance is normalized by the total number of sequences in the cluster, potentially leading to clusters in which most of the sequences are very close together but one sequence is only distantly related to the group. Confirming this prediction, in our longitudinal analysis the genetic distance threshold did not have to be increased in 2007 to capture most 2005 clusters despite the additional of a large number of sequences. Second, maximum genetic distance is a metric more comparable to the time depth used to identify clusters in BEAST [22]. Third, maximum genetic distance is faster to compute, improving program efficiency. We nevertheless plan on adding alternative measures of genetic distance (mean and median) to future releases of the CP. Another difference between the programs is that distances are calculated de-novo from the sequences in the CP, while in PhyloPart, the patristic distances are used. Cluster definition in PhyloPart is a function of the whole tree: a subtree is classified as a cluster if its median genetic distance is smaller than a percentage of the whole tree. However, the user-specified genetic distance threshold in the CP allows external information to be incorporated into the definition, such as the average observed distance within transmission pairs if that is available. We chose this strategy because it is the most widely used definition; in fact, previous studies have demonstrated epidemiologically related viral sequences had less than 4.8% nucleotide substitutions between them [3]. Similarly, because studies vary in the bootstraps they use for support of clusters, we left this as a flexible option for the user to choose.

For data sets containing up to 17,000 sequences, both PhyloPart and CP yield results on a desktop in reasonable time. Theoretically, PhyloPart will slow down in large datasets, as it calculates all pairwise distances then stores them, so they can be accessed each time they are needed. This is an advantage for smaller datasets and speeds up processing, but for large trees, the time to generate matrices of all pairwise distances increases as a polynomial function of the number of sequences n (n (n-1)/2 computations). The CP calculates pairwise genetic distances within a potential cluster as required even if those distances were already calculated when the parent node was tested (and rejected). Nevertheless, the CP was not slower than PhyloPart on small datasets and in fact completed on average three times more rapidly. On large trees, it becomes faster to calculate subsets of pairwise genetic distances only within potential clusters, even if this must be repeated several times. Another alternative, not explored here, is the single-linkage approach proposed by Wertheim et al [34], which does not require a phylogenetic tree and calculates pairwise distances only once. With expanding sizes of HIV-1 data sets and other fast evolving pathogens, there is increasing need for new faster algorithms.

## Conclusions

Our longitudinal cluster analysis demonstrated differences in cluster growth between clusters that were confined to single UK locations in 2005, and those that already contained sequences from several locations across the UK. If confirmed, these results suggest that targeting interventions on individuals within multiple origin clusters to prevent onward transmission would yield disproportionate results. Such real-time analyses are made possible by the CP and CM. As our purpose here was to demonstrate the functionality of the CP and CM, we chose a simple example. We hope that others will use the tools in more elaborate ways to truly provide insight into the dynamics of HIV transmission, as well as other infectious diseases. The CM, for example, can easily be used to compare clusters between trees built with different methods. Concerning cluster dynamics, we note that new sequences added to clusters do not necessarily reflect new infections: they could reflect new diagnoses within the time frame, and one potential explanation of the observed cluster growth may indeed be referral-based testing.

The automation of cluster picking and matching with epidemiological information is a necessary advance as pathogen sequence databases have become too large to analyze manually. The *pol* region of HIV is routinely sequenced for clinical purposes, and several European countries have created central repositories for the sequences. These data, combined with the tools we have made available, offer opportunities for the real-time surveillance of the HIV epidemic. We hope that by providing strategies for cluster identification and description, these user-friendly tools will facilitate comparisons of epidemics between studies and countries.

## Availability and requirements

**Project name:** HIV Clusters in Phylogenetic Trees

**Project home page:**http://hiv.bio.ed.ac.uk/software.html

**Operating system(s):** Platform independent

**Programming language:** Java

**Other requirements:** Java 1.6.0 or higher

**License:** GNU GPLv3

**Any restrictions to use by non-academics:** no restriction

## Abbreviations

CP: Cluster Picker; CM: Cluster Matcher; HIV: Human immunodeficiency virus; HIVRDB: UK HIV drug resistance database; UK: United Kingdom.

## Competing interests

The authors declare that they have no competing interests.

## Authors’ contributions

MRC carried out the clustering analysis, comparative software testing and drafted the manuscript. SJL and EH designed and wrote the software. MRC and EH wrote the software tutorials, and SH and EF independently tested the software. SJL, EH, MRC, SH and AJLB participated in the initial design of the software. AJLB, MRC, SH, EF and VD participated in the data collection and interpretation of clustering results. AJLB, MRC, SJL and EH conceived of the study, participated in its design and coordination and helped to draft the manuscript. All authors read and approved the final manuscript.

## Supplementary Material

Additional file 1 Cluster Picker analysis of hepatitis C virus, avian influenza, pandemic influenza and H3N2 seasonal influenza. Click here for file

Additional file 2 R scripts for linking Cluster Picker and Cluster Matcher outputs and comparing growth of single vs. multiple origin clusters. Click here for file

Additional file 3: Figure S3 Largest UK cluster in 2007. This cluster had 17 sequences in 2007 and 33 in 2007. In 2005, the cluster contained sequences from four different regions (regions 1-4). Region U indicates a sequence from an unknown location. This figure was produced using the Cluster Matcher. Click here for file

Additional file 4 Python script to launch PhyloPart in a loop. Click here for file

Additional file 5: Figure S5 Time to Completion and Computational Complexity. The time to completion (in seconds) of the Cluster Picker on subsamples of HIV dataset (see Table 1) is well approximated by f(N2) (N=number of sequences), indicating a computational complexity of O(N2). Click here for file
