# Supplementary material for: Automated analysis of phylogenetic clusters
Source: BMC Bioinformatics. 2013 Nov 6;14:317. doi: 10.1186/1471-2105-14-317 (PMC4228337; doi:10.1186/1471-2105-14-317)
Supplement: Additional file 1 — Cluster Picker analysis of hepatitis C virus, avian influenza, pandemic influenza and H3N2 seasonal influenza. [file 1471-2105-14-317-S1.pdf]

Additional Data Sets

All input and output files for the runs in this document can be downloaded from our website http://hiv.bio.ed.ac.uk/software.html.

Hepatitis C virus

Previous phylogenetic analyses of Hepatitis C virus (HCV) have used different regions of the genome, including E1, E2 and the HCV core region. We selected the core region because it is longest (~500 nucleotides). Core sequences (>200 nucl.) were downloaded from the Los Alamos National Laboratories repository (http://hcv.lanl.gov). Sequences were sorted by subtype, and as HCV subtype 1b was the most frequent, it was chosen for analysis. After deletion of duplicates, 3786 sequences remained, with a mean sequence length of 505 nucleotides (accession numbers listed in Additional File 5). Accompanying information was downloaded in parallel, including country of origin for 88% of sequences. Sequences originated from 46 different countries. A phylogenetic tree with 100 bootstrap replicates was reconstructed using RaxML [1]. Based on previous work, a cluster threshold of 70% bootstrap and 2% genetic distance was used to identify clusters [2]. The Cluster Picker (CP) completed in <6 seconds, and identified 144 clusters. When the bootstrap was increased to 90%, only 50 clusters were identified. Using the Cluster Matcher (CM), we determined that at both thresholds, sequences from the same country clustered together 100% of the time, suggesting that they represent epidemiologically-relevant clusters. Note that multiple sequences may originate from the same patient and that the tree is fully bifurcating.

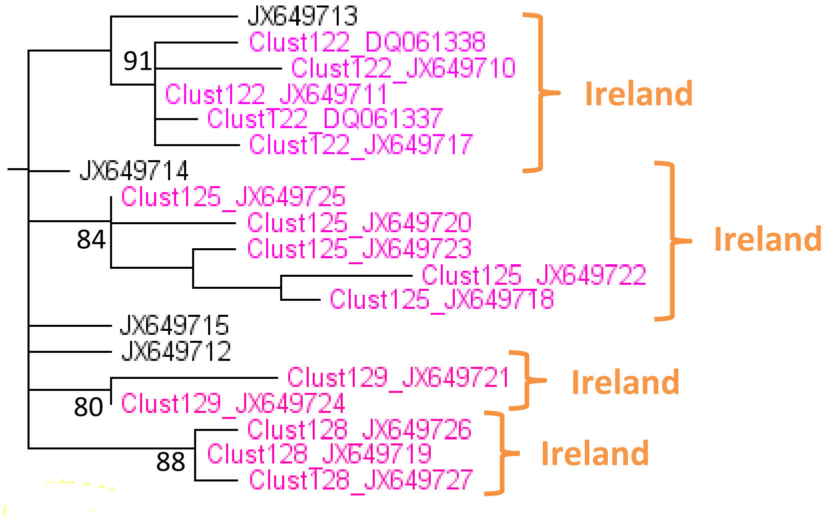

Figure S1.1: Example HCV clusters showing clustering of Irish sequences.

Avian Influenza A

The identification of transmission clusters is irrelevant in flu as the virus is transmitted so rapidly and so frequently that it is highly unlikely that transmission pairs will be sampled. Instead, we have used the CP to down sample a dataset of sequences while maintaining its full genetic diversity. This might be useful for downstream analyses in BEAST [3], or to investigate the effects of sampling. In the present analysis, the CP was run with the aim of downsizing the dataset from 2989 sequences to ~150. All PB2 avian influenza A sequences were downloaded from NCBI Influenza Virus Resource [4], duplicate sequences were removed, and a phylogenetic tree reconstructed in FastTree. The CP was run with a series of thresholds (using the command line version) until we found a threshold which maximized inclusion of sequences in ~150 clusters. 70% bootstrap and 6% genetic distance thresholds yielded 158 clusters containing 98% of sequences. A single sequence from each cluster was selected for further analysis (L. Lu, *manuscript in preparation*).

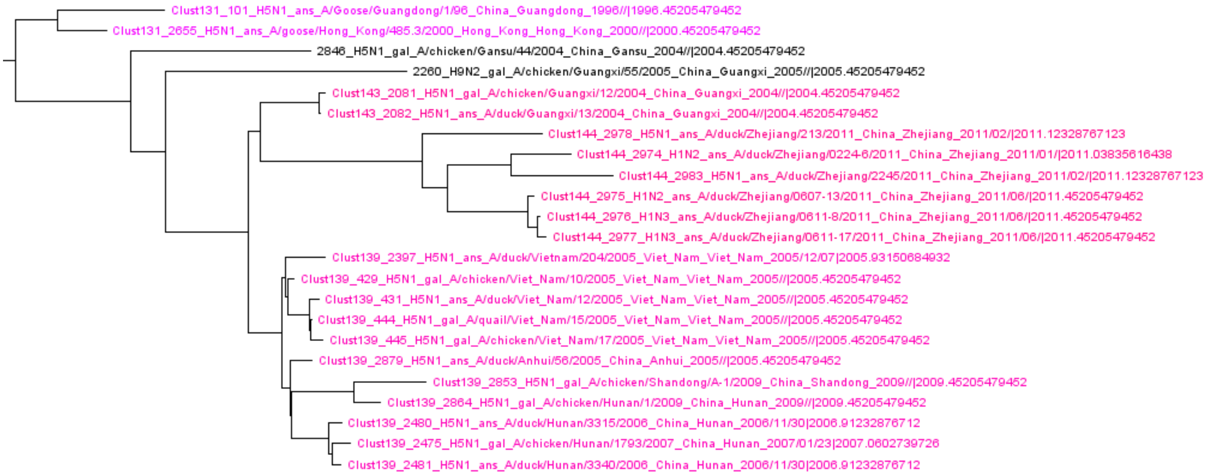

Figure S1.2: Example Avian Influenza clusters

Pandemic Influenza

We used the CP in a BEAST Maximum Clade Credibility (MCC) [3] tree comprising 492 full genome H1N1 human pandemic sequences (segments concatenated, excluding PB1), mostly from the Spring and Fall waves of the pandemic (2009-2010). Details of the sequences and BEAST tree models can be found in the original publication [5]. The BEAST MCC tree was generated with Tree Annotator (distributed with BEAST) in the extended nexus format, and was converted into a newick format with the posterior probabilities encoded as support values at the nodes using an R script MCC_to_NWK.R (see the Tutorial files). Initial and main support thresholds of 0.7 were used together with a genetic distance threshold of 2%. These resulted in three large clusters, broadly corresponding to the initial clades present in the USA at the start of the pandemic as determined by hand on a maximum likelihood tree on USA only data [6]. The largest of these clusters (Cluster3, purple) corresponds to “Global Clade 7”, which became dominant in Wave 2 of the pandemic.

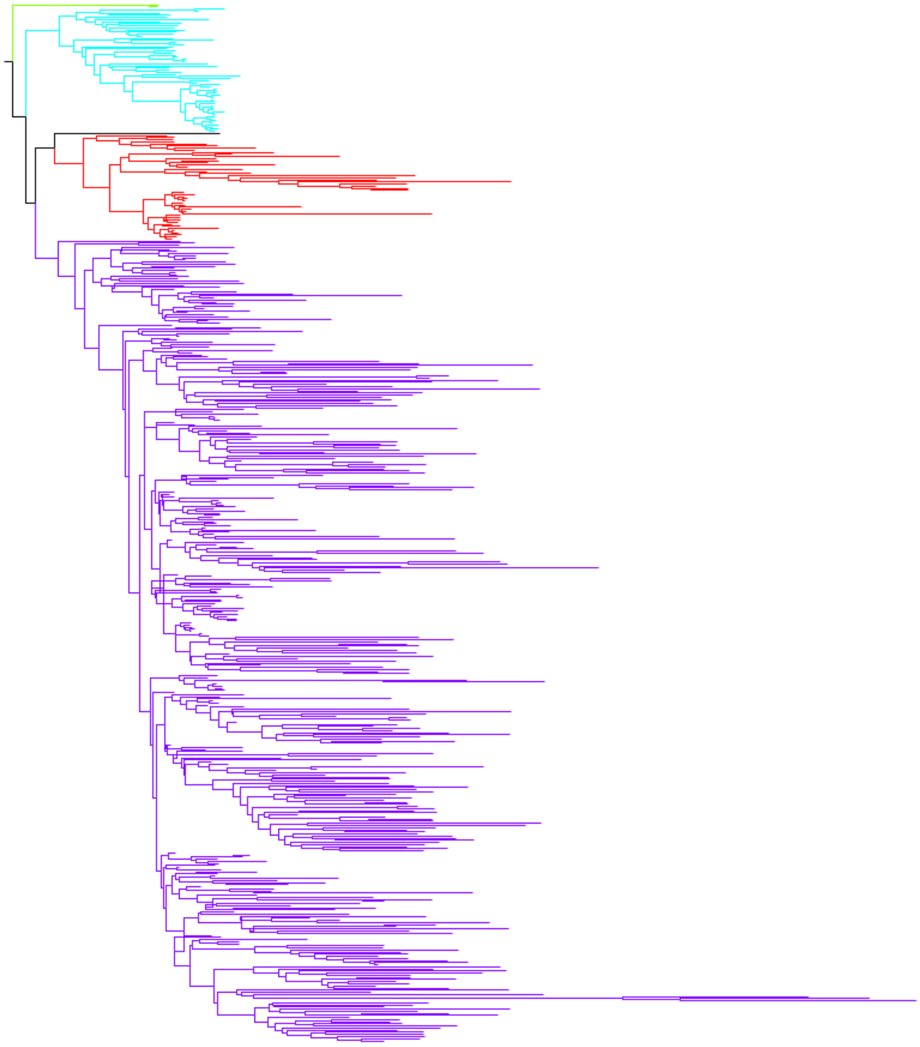

Figure S1.3: Time Resolved BEAST MCC Tree of H1N1 Pandemic Sequences, colored by cluster using the CP. Cyan: Cluster2 (59 sequences), Purple: Cluster3 (380 sequences, “Global Clade 7”), Red: Cluster4 (50 sequences).

H3N2 Seasonal Influenza

A data set comprising 1027 H3N2 human seasonal influenza full length haemagglutinin sequences, from 2004 – 2012 was obtained from the NCBI Influenza Virus Resource [4]. Sequences from each isolate were classified by global region (North, Tropical and South), continent and year, similar to the H3N2 Global Migration Dynamics study by Bedford et al [7]. Time resolved trees were generated using BEAST with the SRD06 nucleotide model, the relaxed lognormal uncorrelated clock and constant population size. Two independent runs of 500,000,000 MCMC samples (step 10,000) were combined, and the final MCC tree was converted to newick format (using MCC_to_NWK.R). The CP was used with initial and main support thresholds of 0.7, and genetic distance threshold of 2% to reveal 93 clusters, including 76% of the sequences. The composition of the clusters was analyzed with the CM. We found that on average 76% of the sequences in a large cluster (>= 20 sequences) were isolated in the same year, 72% of them were isolated in the same global region (North, Tropical, South) but only 66% in the same continent.

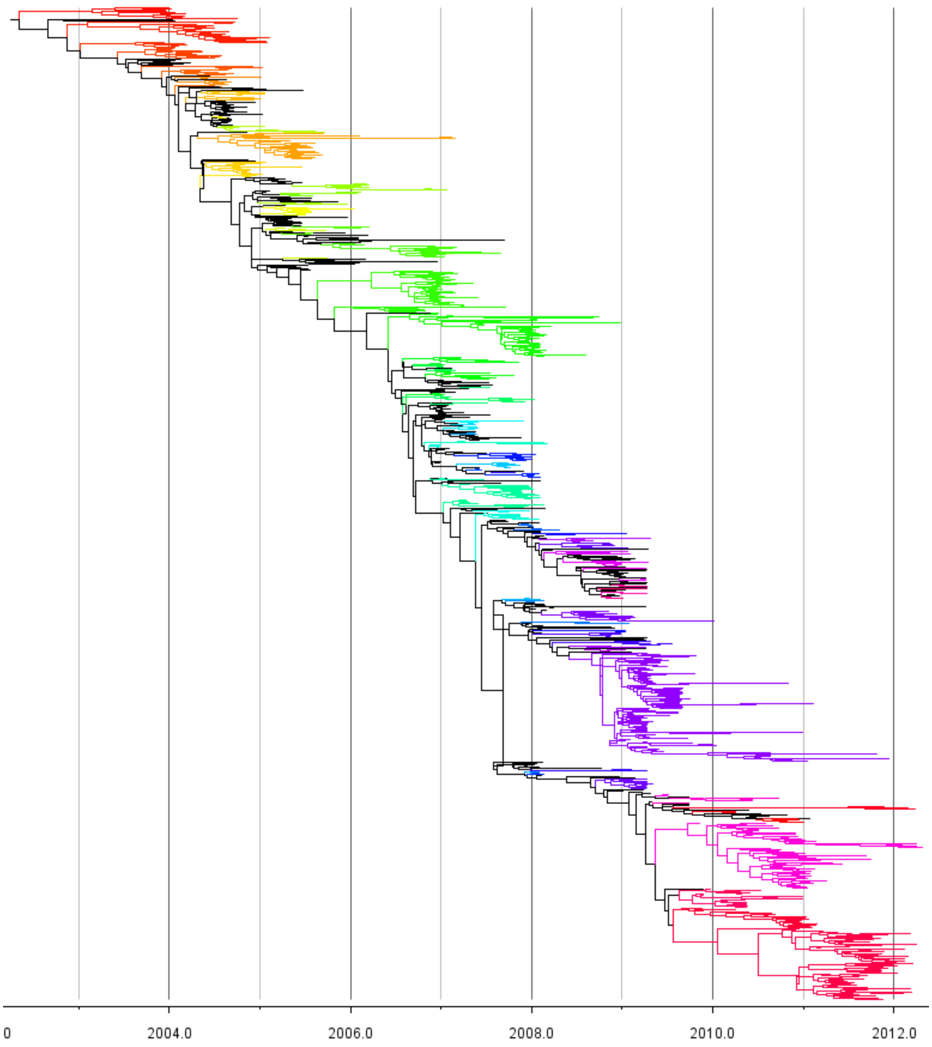

Figure S1.4: Time resolved BEAST MCC tree of H3N2 human seasonal influenza haemagglutinin sequences, split into 93 clusters using the CP.

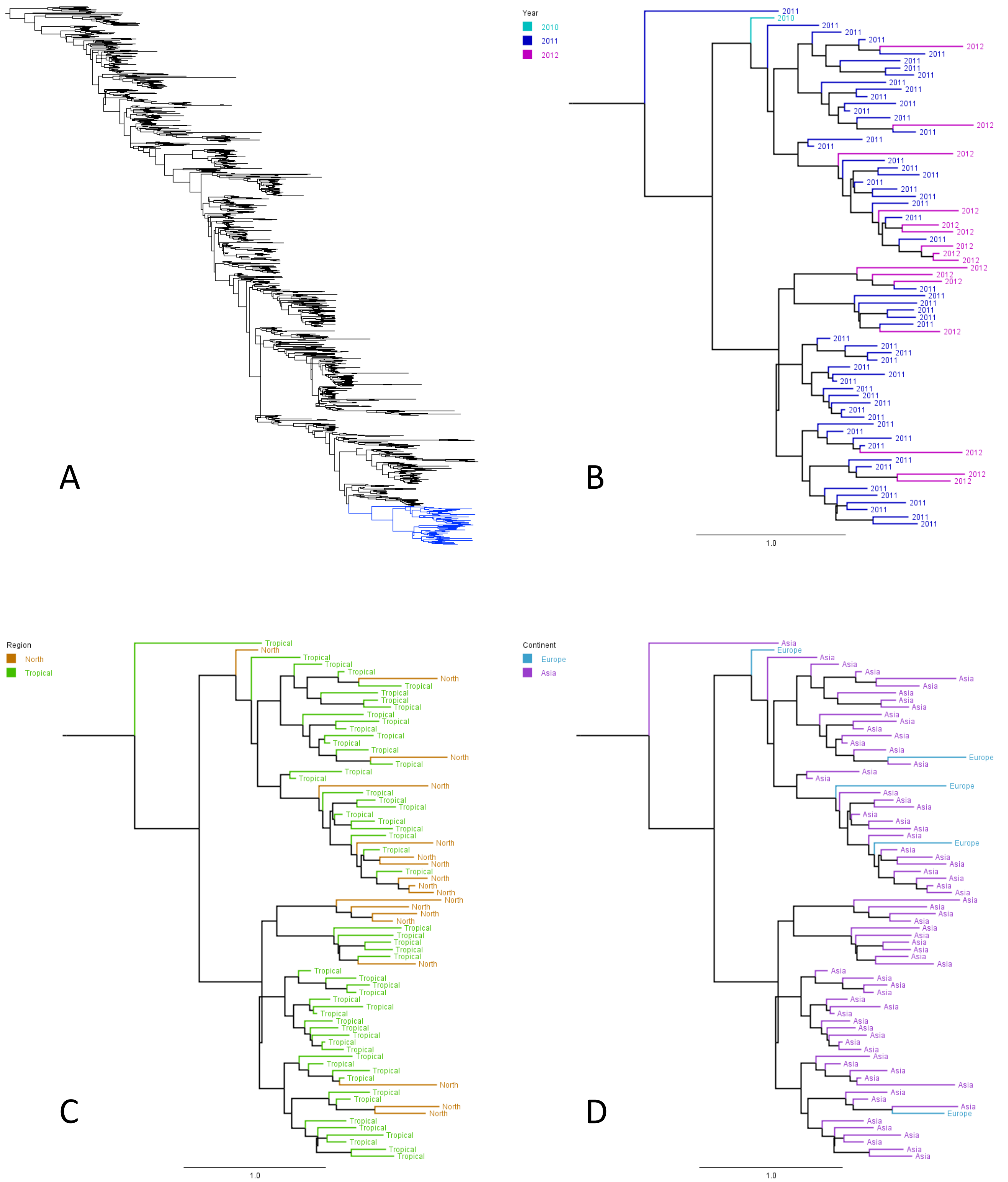

Figure S1.5: A - Example “Cluster 89” selected using the Cluster Matcher on the whole tree (clusters >= 20 sequences, with >=50% from Asia). B – zoomed “Cluster 89” colored by year of isolation, showing that most of the sequences are from 2011/2012. “Cluster 89” colored by global region (C), and by continent (D), showing that most of the sequences in this cluster were circulating in southern Asia (Tropical + Asia) with later spill over into Europe. Coloring was performed using the CM.

Reference List

1. Stamatakis A: **RAxML-VI-HPC: maximum likelihood-based phylogenetic analyses with thousands of taxa and mixed models**. *Bioinformatics* 2006, **22:**2688-2690.

2. Sacks-Davis R, Daraganova G, Aitken C, Higgs P, Tracy L, Bowden S, Jenkinson R, Rolls D, Pattison P, Robins G, Grebely J, Barry A, Hellard M: **Hepatitis C virus phylogenetic clustering is associated with the social-injecting network in a cohort of people who inject drugs**. *PLoS ONE* 2012, **7:**e47335.

3. Drummond AJ, Rambaut A: **BEAST: Bayesian evolutionary analysis by sampling trees**. *BMC Evol Biol* 2007, **7:**214.

4. Bao Y, Bolotov P, Dernovoy D, Kiryutin B, Zaslavsky L, Tatusova T, Ostell J, Lipman D: **The influenza virus resource at the National Center for Biotechnology Information**. *J Virol* 2008, **82:**596-601.

5. Lycett S, McLeish NJ, Robertson C, Carman W, Baillie G, McMenamin J, Rambaut A, Simmonds P, Woolhouse M, Leigh Brown AJ: **Origin and fate of A/H1N1 influenza in Scotland during 2009**. *J Gen Virol* 2012, **93:**1253-1260.

6. Nelson MI, Tan Y, Ghedin E, Wentworth DE, St GK, Edelman L, Beck ET, Fan J, Lam TT, Kumar S, Spiro DJ, Simonsen L, Viboud C, Holmes EC, Henrickson KJ, Musser JM: **Phylogeography of the spring and fall waves of the H1N1/09 pandemic influenza virus in the United States**. *J Virol* 2011, **85:**828-834.

7. Bedford T, Cobey S, Beerli P, Pascual M: **Global migration dynamics underlie evolution and persistence of human influenza A (H3N2)**. *PLoS Pathog* 2010, **6:**e1000918.
