# Supplementary material for: Automated analysis of phylogenetic clusters
Source: BMC Bioinformatics. 2013 Nov 6;14:317. doi: 10.1186/1471-2105-14-317 (PMC4228337; doi:10.1186/1471-2105-14-317)

Figure S3. Largest UK cluster in 2007. This cluster had 17 sequences in 2007 and 33 in 2007. In 2005, the cluster contained sequences from four different regions (regions 1-4). Region U indicates a sequence from an unknown location. This figure was produced using the Cluster Matcher.

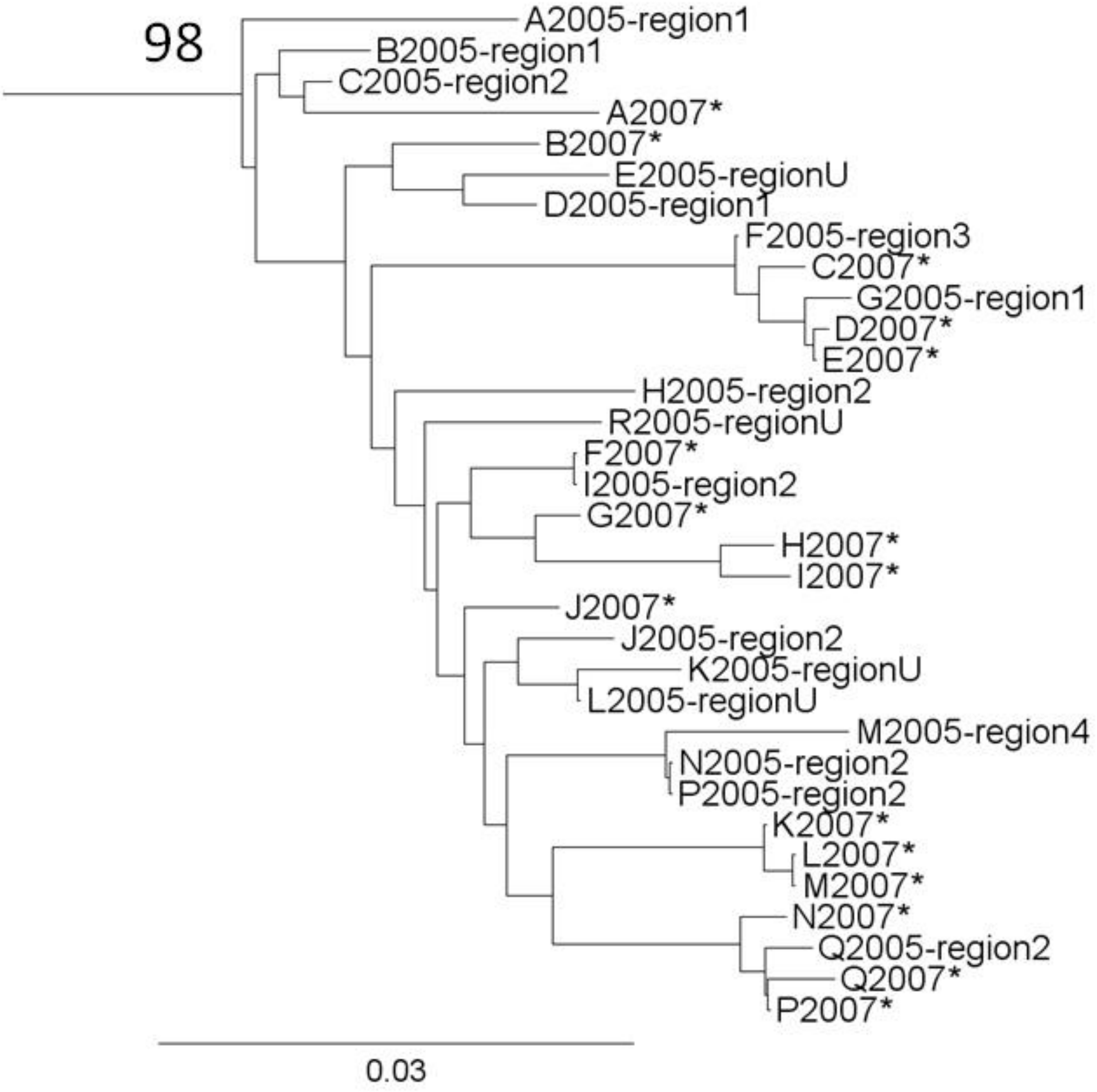

Supplement: Additional file 3: Figure S3 — Largest UK cluster in 2007. This cluster had 17 sequences in 2007 and 33 in 2007. In 2005, the cluster contained sequences from four different regions (regions 1-4). Region U indicates a sequence from an unknown location. This figure was produced using the Cluster Matcher. [file 1471-2105-14-317-S3.pdf]
