# Supplementary material for: Automated analysis of phylogenetic clusters
Source: BMC Bioinformatics. 2013 Nov 6;14:317. doi: 10.1186/1471-2105-14-317 (PMC4228337; doi:10.1186/1471-2105-14-317)

Figure S5 . Time to Completion and Computational Complexity. The time to completion (in seconds) of the Cluster Picker on subsamples of HIV dataset (see table 1) is well approximated by $f(N^2)$ (N=number of sequences), indicating a computational complexity of $O(N^2)$

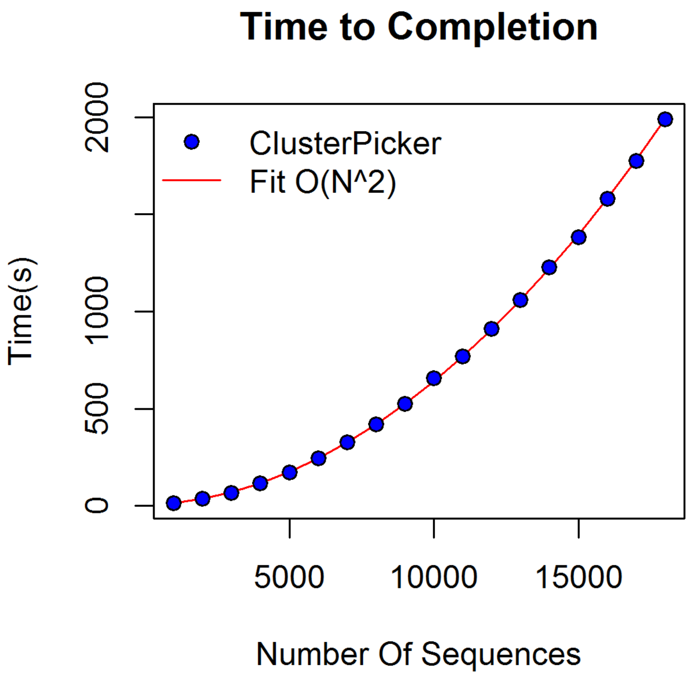

Supplement: Additional file 5: Figure S5 — Time to Completion and Computational Complexity. The time to completion (in seconds) of the Cluster Picker on subsamples of HIV dataset (see Table 1) is well approximated by f(N2) (N=number of sequences), indicating a computational complexity of O(N2). [file 1471-2105-14-317-S5.pdf]
